# Supplementary material for: Urea Fertilization Significantly Promotes Nitrous Oxide Emissions from Agricultural Soils and Is Attributed to the Short-Term Suppression of Nitrite-Oxidizing Bacteria during Urea Hydrolysis
Source: Microorganisms. 2024 Mar 28;12(4):685. doi: 10.3390/microorganisms12040685 (PMC11052285; doi:10.3390/microorganisms12040685)
Supplement: Supplementary file 1 [file microorganisms-12-00685-s001.zip › microorganisms-2930650-supplementary.pdf]

Table S1. Characteristics of the four soils tested in the study. The value in the parenthesis stands for the standard deviation obtained for 3 replicates.

Soil name	Soil texture class①	Bulk density (g cm^{-3})②	Water content (w/w)③	pH④	Initial N-NH_4^+⑤ (μg N g^{-1} soil)	Initial N-NO_3^-⑥ (μg N g^{-1} soil)	Total organic carbon content⑦
Solanum	Sandy Loam	1.26	0.23 (0.025)	6.83 (0.02)	0.74 (0.08)	79.46 (0.97)	1.1% (0.08)
Lettuce	Clay	1.38	0.22 (0.013)	5.25 (0.03)	0.94 (0.12)	54.08 (2.89)	1.4% (0.10)
Banana	Sandy Loam	1.29	0.18 (0.020)	6.35 (0.03)	9.68 (0.22)	9.61(1.36)	2.0% (0.07)
Pitaya	Clay	1.30	0.29 (0.005)	5.58(0.01)	12.36 (0.89)	12.05 (2.23)	2.3% (0.14)

① SRUC, Craibstone, Scotland; grid reference NJ872104. ② Reference Kemp et al (*Biol. Fert. Soils* **1992**, *13(4)*, 218-224, doi: 10.1007/BF00340579). ③ Oven drying method. ④ Measured in 1:5 soil:1 M KCl extracts. ⑤ Indophenol Blue method, measured in 1:5 soil:2g L^{-1} $CaSO_4$ extracts. ⑥ Ion chromatography, measured in 1:5 soil:2g L^{-1} $CaSO_4$ extracts. ⑦ Reference standard HJ 501-2009.

Table S2. Soil pH after 0, 12, 24, 36, 48 and 72 hours of first microcosm experiment.

Soil	Applied Fertilizer (μg N g^{-1} $soil_{dw}$)	pH					
		0 h	12 h	24 h	36 h	48 h	72 h
Solanum	NH_4^+-250	6.70	6.58	6.43	6.30	6.13	5.76
	Urea-250	6.85	7.14	7.22	7.07	6.78	6.15

Measured in 1:5 soil:1 M KCl extracts.

Table S3. Soil pH after 0, 3, 6 and 9 days of second microcosm experiment.

Soil	Applied Fertilizer (μg N g^{-1} $soil_{dw}$)	pH			
		0 days	3 days	6 days	9 days
Solanum	NH_4^+-100	6.81	5.95	6.03	6.01
	NH_4^+-250	6.70	5.76	5.48	5.46
	NH_4^+-450	6.64	5.70	5.36	5.23
	Urea-100	6.84	6.15	6.20	6.25
	Urea-250	6.85	6.14	5.92	5.90
	Urea-450	6.84	6.37	5.74	5.54

Measured in 1:5 soil:1 M KCl extracts.

Table S4. Soil pH after 0, 3, 6 and 9 days of third microcosm experiment.

Soil	Applied Fertilizer (μg N g^{-1} $soil_{dw}$)	pH			
		0 days	3 days	6 days	9 days
Solanum	NH_4^+ (CK)	6.70	5.76	5.48	5.46
	NH_4^++ 10 ug g^{-1} $soil_{dw}$ $KClO_3$	6.70	6.41	6.10	6.01
	NH_4^++ 30 ug g^{-1} $soil_{dw}$ $KClO_3$	6.70	6.45	6.19	6.05
	Urea (CK)	6.85	6.14	5.92	5.90
	Urea + 6.4 ug g^{-1} $soil_{dw}$ NBPT	6.85	6.12	5.93	5.87
	Urea + 12.8 ug g^{-1} $soil_{dw}$ NBPT	6.85	6.21	6.08	5.90

Measured in 1:5 soil:1 M KCl extracts.

Table S5. Nitrite oxidation potential after 3, 12, 24, 48 hours of the incubation of FA.

Soil	Ammonia in head-space (v/v)	Nitrite oxidation potential (μmol g^{-1} $soil_{dw}$ h^{-1})			
		3h	12h	24h	48h
Solanum	CK	0.248^{aa}	0.253^{aa}	0.257^{aa}	0.256^{aa}
	0.9%	0.238^{aa}	0.215^{bb}	0.206^{cb}	0.192^{db}
	2.7%	0.236^{aa}	0.209^{bb}	0.190^{bc}	0.120^{cc}
	4.5%	0.224^{ab}	0.192^{bc}	0.164^{cd}	0.105^{dc}
Lettuce	CK	0.141^{aa}	0.145^{aa}	0.140^{aa}	0.134^{aa}
	0.9%	0.140^{aa}	0.129^{bb}	0.087^{cb}	0.057^{cb}
	2.7%	0.137^{aa}	0.123^{bb}	0.062^{cc}	0.025^{cc}
	4.5%	0.132^{aa}	0.110^{bc}	0.043^{cc}	0.015^{dc}
Banana	CK	0.331^{aa}	0.330^{aa}	0.334^{aa}	0.331^{aa}
	0.9%	0.331^{aa}	0.316^{bb}	0.311^{bb}	0.312^{bb}
	2.7%	0.328^{aa}	0.311^{bc}	0.282^{cc}	0.268^{cc}
	4.5%	0.325^{aa}	0.308^{bc}	0.271^{dc}	0.246^{cd}
Pitaya	CK	0.198^{aa}	0.192^{aa}	0.194^{aa}	0.197^{aa}
	0.9%	0.194^{aa}	0.183^{ba}	0.163^{cb}	0.153^{cb}
	2.7%	0.180^{ab}	0.156^{bb}	0.124^{cc}	0.109^{dc}
	4.5%	0.180^{ab}	0.148^{bb}	0.117^{cc}	0.073^{dd}

One-way ANVOD was performed using Duncan's method, with different first letters in the same row and different second letters in the same column indicating significant differences ($p < 0.05$).

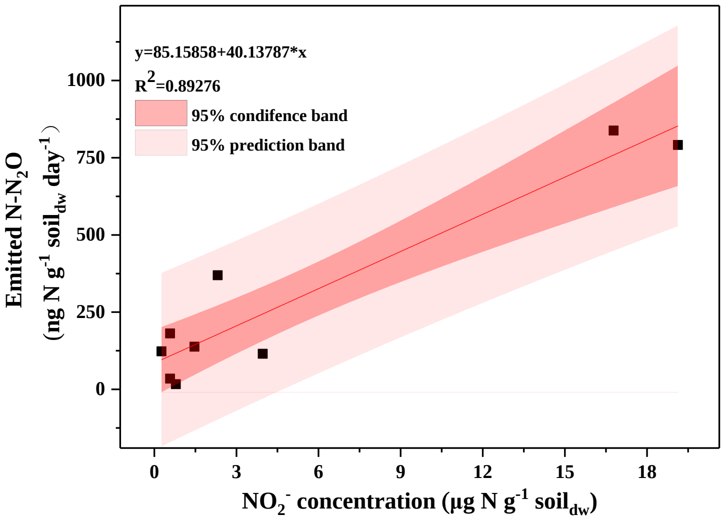

Figure S1. Correlation analysis between N_2O emission rate and NO_2^- concentration in microcosm experiment.

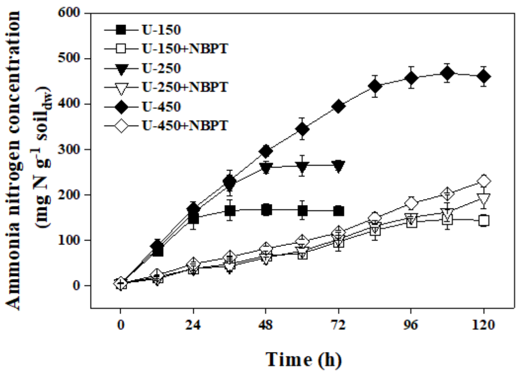

(**a**)

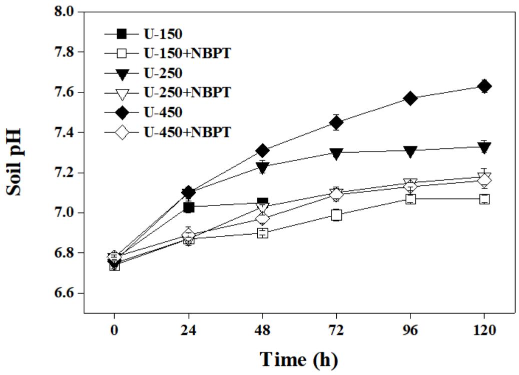

(**b**)

Figure S2. (a) Hydrolysis of urea; (b) changes of soil pH in microcosm. Hydrolysis reactions operated in soil microcosm with acetylene to inhibit AOM. The concentration of NBPT was 6.4 μg g^{-1} $soil_{dw}$.

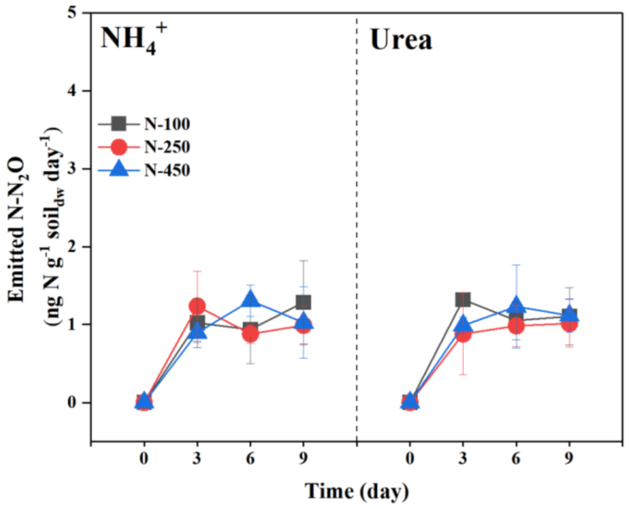

Figure S3. Changes of N_2O emission rate in second microcosm experiment with AOM inhibitor acetylene.

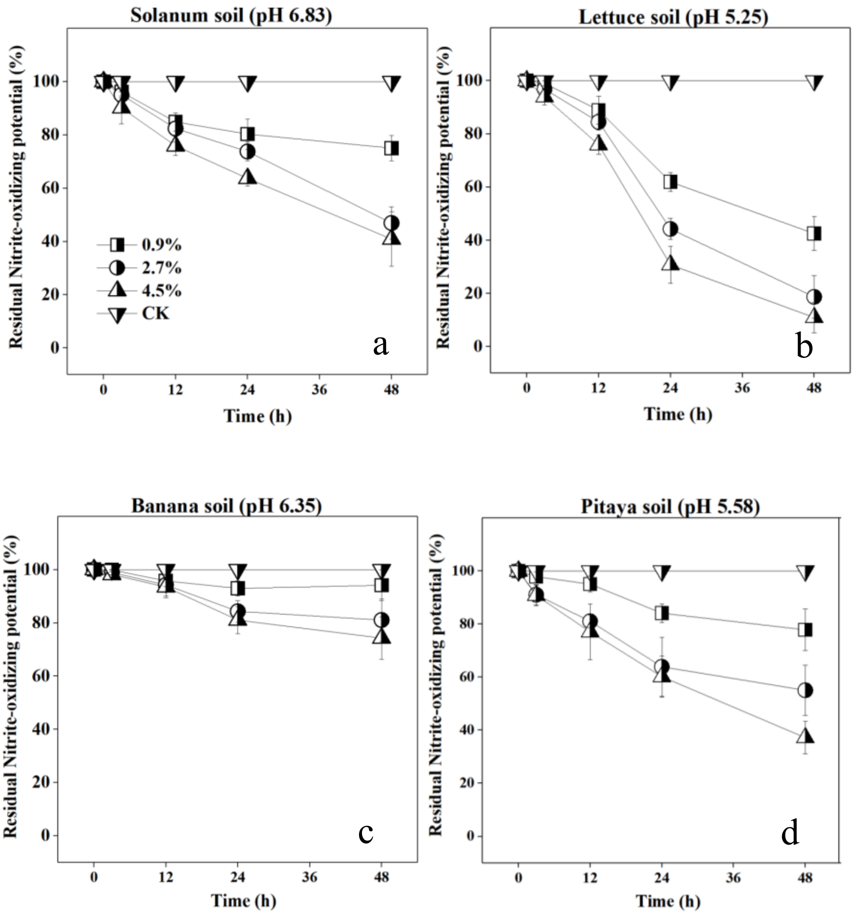

Figure S4. Time-series change of the residual nitrite-oxidizing potential in (a) solanum soil, (b) lettuce soil, (c) banana soils and (d) pitaya soil when responding to different concentration of FA. CK: without addition of FA.
